# Co-localisation of abnormal brain structure and function in specific language impairment

**DOI:** 10.1016/j.bandl.2011.10.006

**Published:** 2012-03

**Authors:** Nicholas A. Badcock, Dorothy V.M. Bishop, Mervyn J. Hardiman, Johanna G. Barry, Kate E. Watkins

**Affiliations:** a Department of Experimental Psychology, University of Oxford, South Parks Road Oxford OX1 3UD, United Kingdom; b MRC Institute of Hearing Research, University of Nottingham, University Park, Nottingham NG7 2RD, United Kingdom; c FMRIB Centre, University of Oxford, John Radcliffe Hospital, Headley Way, Oxford OX3 9DU, United Kingdom

**Keywords:** Developmental disorders, Voxel-based morphometry, Functional magnetic resonance imaging, Language specialisation, Lateralisation

## Abstract

We assessed the relationship between brain structure and function in 10 individuals with specific language impairment (SLI), compared to six unaffected siblings, and 16 unrelated control participants with typical language. Voxel-based morphometry indicated that grey matter in the SLI group, relative to controls, was increased in the left inferior frontal cortex and decreased in the right caudate nucleus and superior temporal cortex bilaterally. The unaffected siblings also showed reduced grey matter in the caudate nucleus relative to controls. In an auditory covert naming task, the SLI group showed reduced activation in the left inferior frontal cortex, right putamen, and in the superior temporal cortex bilaterally. Despite spatially coincident structural and functional abnormalities in frontal and temporal areas, the relationships between structure and function in these regions were different. These findings suggest multiple structural and functional abnormalities in SLI that are differently associated with receptive and expressive language processing.

## Graphical abstract

**Graphical abstract:**

**Figure:**
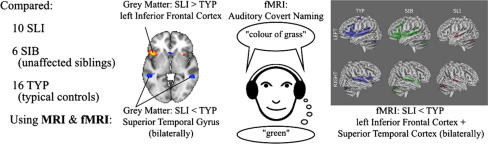

**Highlights:**

► Compared brain structure and function in SLI, unaffected siblings, and typical peers. ► More grey matter in SLI in the left inferior frontal cortex. ► Less grey matter in SLI in the right caudate nucleus and superior temporal cortex bilaterally. ► Functional activation was examined with an auditory covert naming task. ► Functionally, SLI had reduced activation of the left inferior frontal cortex and the superior temporal cortex bilaterally.

## 1 Introduction

Specific language impairment (SLI) is a developmental disorder affecting of 2–7% of the population (Law, Boyle, Harris, Harkness, & Nye, 1998; Tomblin et al., 1997). It is diagnosed on the basis of difficulties with the production and reception of language in a child who is otherwise developing normally. The disorder is highly heritable (Bishop, 2002) but usually the patterns of inheritance are complex and likely due to multiple and interacting genetic and environmental risk factors (see Bishop, 2009 for a recent review). The search for neural correlates of language impairment in developmental disorders like SLI has provided rather mixed results. This is partly due to rapid advances in non-invasive methodologies to study brain structure and function that have outpaced data collection; it is rare that any two studies have implemented the same methods. In addition, previous work has focused on using brain imaging to differentiate between developmental disorders such as dyslexia and SLI. A clearer picture of the brain abnormalities associated with SLI will contribute to our understanding of the neurobiological phenotype and may ultimately aid genetic analyses.

Previous investigations of brain structure in SLI have focused on peri-Sylvian cortical language areas and the asymmetry of these structures. In the anterior language cortex (inferior frontal gyrus or Broca’s area), abnormal gyrification (Clark & Plante, 1998; Cohen, Campbell, & Yaghmai, 1989), reduced volume (Gauger, Lombardino, & Leonard, 1997), and atypical rightward asymmetry (De Fossé et al., 2004) have been described in language-impaired children and adults. Atypical rightward asymmetry is also described in SLI in the posterior language cortex (Herbert et al., 2005; Jernigan, Hesselink, Sowell, & Tallal, 1991), including posterior peri-Sylvian areas (Plante, Swisher, Vance, & Rapcsak, 1991) and the planum temporale specifically (Gauger et al., 1997; but see Preis, Jäncke, Schittler, Huang, & Steinmetz, 1998). These studies suggest that abnormal brain development, possibly of a genetic aetiology, results in atypical structural asymmetries that in turn give rise to abnormal functional organisation.

Consistent with this notion, studies of the functional organisation of language in SLI suggest weak language skills are associated with departures from the normal pattern of left-hemisphere specialisation for language. The first studies to investigate this question used single-photon emission computed tomography (SPECT) to measure regional cerebral blood flow. Three studies measured blood flow at rest and found reduced asymmetry, or hypoperfusion of the left hemisphere, or both in language-impaired children compared to controls (Denays et al., 1989; Lou, Henriksen, & Bruhn, 1990; Ors et al., 2005). A further SPECT study used a dichotic listening task to activate language areas, and found less left hemisphere activation in children with language problems compared to controls (Chiron et al., 1999). Two subsequent studies using functional magnetic resonance imaging (fMRI) did not find convincing lateralisation differences between cases with SLI versus controls, but they used activation tasks that did not give substantial hemispheric differences in the control group (Ellis Weismer, Plante, Jones, & Tomblin, 2005; Hugdahl et al., 2004). One fMRI study used listening to a recording of the mother’s voice to successfully activate the left hemisphere in 10 of 14 controls, and whereas right hemisphere activation was seen in 5 of 6 late talkers over the age of 3 years (Bernal & Altman, 2003). Further evidence of atypical cerebral lateralisation was found by Whitehouse and Bishop (2008), who used functional transcranial Doppler ultrasound to measure lateralised blood flow during a word generation task. They found that either symmetrical responses or right hemisphere bias were significantly more common in adults with persistent language impairment than in controls. There is, then, growing evidence of atypical lateralisation of brain responses in language tasks, but only a handful of relevant studies have been conducted. Also, to our knowledge, none have related abnormal functional organisation to brain structural abnormalities in SLI.

An exception is studies of the KE family, where researchers have found related abnormalities in brain structure and function in affected family members (see Vargha-Khadem, Gadian, Copp, & Mishkin, 2005). Speech and language impairment in this family is associated with a mutation of the FOXP2 gene, and shows an autosomal dominant pattern of inheritance (Lai, Fisher, Hurst, Vargha-Khadem, & Monaco, 2001). They cannot therefore be regarded as typical cases of SLI, though many features of their phenotype resemble features seen in SLI. Affected members of the KE family have a verbal dyspraxia evident on tests of nonword repetition and oromotor praxis. In addition, as a group they show impairments on many other tests of language and, in some cases, nonverbal cognition (Watkins, Dronkers, & Vargha-Khadem, 2002a). Imaging studies reveal reduced volume of the caudate nucleus, increased grey matter in the left inferior frontal gyrus and posterior temporal cortex in affected family members (Belton, Salmond, Watkins, Vargha-Khadem, & Gadian, 2003; Watkins et al., 2002b). Functionally, the caudate nucleus is overactive during speech production (Watkins, Gadian, & Vargha-Khadem, 1999), whereas the left inferior frontal gyrus and posterior language cortex are underactive (Liégeois et al., 2003). The volume of the caudate nucleus was found to correlate with performance on tests of nonword repetition and oral praxis (Watkins et al., 2002b). Given the phenotypic similarities between the KE family and more typical SLI, it is of interest to compare brain structure and function of these groups.

Here, we used magnetic resonance imaging (MRI) to investigate brain structure and function in a group of 10 individuals with SLI ranging in age from 8 to 17 years. We compared their data with that obtained in six unaffected siblings, who tended to be older than the SLI group (age range 12–22 years), and a group of 16 unrelated controls with typical language development who were matched in age as closely as possible to the participants from the SLI group and their siblings (6–25 years). Three of the SLI group and two of the unaffected siblings and unrelated control groups were left-handed; all other participants were right-handed. The task used for the fMRI scan was a modified version of an auditory response-naming task (Bookheimer et al., 1998) that reliably activates left inferior frontal cortex (Broca’s area) and posterior superior temporal cortex (Wernicke’s area). The aims of the study were to characterise the brain abnormalities associated with SLI and to determine whether previously described functional and structural abnormalities were related.

## 2 Materials and methods

### 2.1 Participants

Participants were recruited through a research participant database of families with at least one child with SLI and families with typically-developing children who had participated in previous studies (Barry, Yasin, & Bishop, 2007; Barry et al., 2008). Participants were required to have normal hearing (a bilateral pure tone audiometric screening test at 25 db HL ISO for 500, 1000, and 2000 Hz), a non-verbal IQ (NVIQ) score of 80 or above on the Wechsler Abbreviated Scale of Intelligence (Wechsler & Chen, 1999), English as their first language, and no reported neurological impairments. The children with SLI were initially recruited from schools, where they were diagnosed with language learning difficulty. SLI group membership was based upon performance below the 10th percentile on two or more standardised tests of language or literacy ability (note: none of the SLI individuals were included based upon two low literacy scores alone). Typically developing individuals had no reported history of language or literacy problems and scored above the 10th percentile on all standardised tests of language or literacy ability.

Images of brain structure were obtained in 10 individuals with SLI, 6 individuals with typical language skills who were siblings of individuals with SLI (Siblings or SIB), and 16 individuals with typical language skills with no family history of SLI (Typical or TYP). We were unable to obtain additional functional imaging data from two children with SLI and three children from the Typical group. Descriptive statistics for age, non-verbal IQ, gender, handedness, and behavioural performance measures (see below) for each of the participants are presented along with group medians in Table 1. These indicate that the SLI group had both receptive and expressive language difficulties, as well as poor literacy skills. Their very low scores on oromotor sequences and nonword repetition indicate difficulties in programming or remembering sequences of speech sounds, even when no meaning was involved.

### 2.2 Materials

The psychometric assessment battery included tests of non-verbal reasoning, understanding of grammar, reading skills, oromotor coordination, and handedness and took on average 1.5 h to administer.

The block design and matrix reasoning task from the WASI (Wechsler & Chen, 1999) were used to assess non-verbal reasoning skills. Scores were converted into age-scaled scores.

#### 2.2.1 Language/literacy assessments

Parental report of communication skills was assessed with the Children’s Communication Checklist, version-2 (CCC-2; Bishop, 2003a) or the Communication Checklist for Adults (CC-A; Whitehouse & Bishop, 2009) depending on age. These communication checklists were designed to assess strengths and weaknesses in communication, which are not readily identified by traditional language tests, and yield a General Communication Composite (GCC). A GCC score greater than 58 is within the normal range.

The electronic version of Test for Reception of Grammar-2 (TROG-2; Bishop, 2003b) is a multiple choice sentence comprehension test used to assess grammatical understanding in children and adults. Scaled scores were derived using UK test norms.

Reading skills were assessed using form B of the Test Of Word Reading Efficiency (TOWRE; Torgesen, Wagner, & Rashotte, 1999) a speeded test that gives scores for reading of real words (sight word reading efficiency) and non-words (phonemic decoding efficiency). Raw scores were converted to standard scores using American norms.

Oromotor coordination was assessed using the oromotor sequences, sentence repetition, and non-word repetition subtests of the NEuroPSYchology (NEPSY) test battery (Korkman, Bortolini, & Kemp, 1998). The norms cover a maximum age of 12 years, 11 months; therefore, we used data from a larger control sample to convert raw scores to standard scores (see Barry et al., 2007).

#### 2.2.2 Handedness

Handedness was assessed using a brief demonstration hand preference (based upon the Edinburgh Handedness Inventory; Oldfield, 1971). Children were asked to demonstrate how they would perform each of 10 actions; write, draw, throw, use scissors, brush their teeth, cut with a knife, use a spoon, sweep with a broom (upper hand), take the lid of a box, and deal cards. Left, Right, or either (if child indicated both) hand was recorded in each case. The number of right hand preferences was taken as a measure of hand dominance.

### 2.3 Scanning

MRI data were obtained using a 1.5-T Siemens Sonata scanner with a single-channel head coil. Participants wore noise-attenuating headphones and padding was inserted around the head to restrict movement. They watched a DVD of their choice via a mirror on the head coil during acquisition of the structural data. A T1-weighted image was acquired in each participant for structural analysis and image registration (3D FLASH; TR = 12 ms; TE = 5.6 ms; 1 mm isotropic voxels; matrix = 256 × 256 × 208; elliptical sampling; orientation = coronal). One acquisition of this T1-weighted sequence took five minutes. At the end of these five minutes, the image was inspected for motion artefacts and, if necessary, children were reminded to keep still for a further five minutes. Three artefact-free images were successfully acquired in each participant. The first and third images were registered (rigid-body transformation; 6 degrees of freedom; trilinear interpolation) to the second image to correct for movement between acquisitions and summed to create a single T1-weighted image in each participant.

Before the functional task, participants were removed from the scanner for a break if necessary. For the functional scan, whole-head T2∗-weighted echo-planar images (TR = 3s; TE = 50 ms; 120 volumes, 6 min), were acquired. Each volume comprised 35 4-mm axial slices (in-plane resolution 3 mm × 3 mm). Stimuli were presented over MRI compatible headphones (MR Confon: http://www.mr-confon.de) at a comfortable listening level (estimated ∼70 dB). Participants were asked to keep their eyes closed.

#### 2.3.1 Functional task

The task used for functional imaging was based on the Auditory Responsive Naming task previously used with PET (Bookheimer et al., 1998). It was chosen because it was thought to be engaging for children, easy enough for them to comply with and known to produce activation in both posterior and anterior language areas (Wernicke’s and Broca’s area, respectively). In the Speech condition, participants heard simple three-word auditory definitions of a high frequency word and were required to silently generate an appropriate word; for example, ‘wear on head’ > silently generate ‘hat’. As a control for the auditory stimulation, a Reversed Speech condition was included during which the recordings used in the Speech condition were digitally reversed, producing meaningless strings of auditory stimulation and maintaining spectrotemporal complexity (as used by Crinion & Price, 2005). Participants listened passively to stimuli in the Reversed Speech condition. The task was explained verbally by the experimenter before the start of the functional data acquisition to ensure participants understood it and could overtly produce a small set of target stimuli. A short practice was given to the participants inside the scanner immediately before the start of data acquisition. During this practice they heard five stimuli for the Speech condition followed by five stimuli for the Reversed Speech condition. Participants were instructed not to overtly produce the target word because speaking produced head movements during scanning. They were asked instead to “think of the word inside their heads” and keep as still as possible. The practice stimuli were not used again during the functional data acquisition. If the participants were happy to proceed with the task, functional data were acquired. The Speech and Reversed Speech conditions and a baseline condition during which no stimuli occurred were presented in 30-s blocks and repeated four times each in a fixed pseudorandom order so that no condition was presented consecutively. Each 30-s block of the Speech and Reversed Speech conditions comprised six stimuli presented one every 5 s.

#### 2.3.2 Structural image analysis

The T1-weighted structural brain images were analysed with an ‘optimised’ voxel-based morphometry (VBM)-style protocol (Good et al., 2001) within FMRIB’s Software Library (FSL v4.1, www.fmrib.ox.ac.uk/fsl). The skull was stripped from this image using the Brain Extraction Tool (Smith, 2002) and the brain images were segmented to form images representing partial volume estimates of each tissue class (i.e. how much of the signal in each voxel was grey or white matter or cerebrospinal fluid) (Zhang, Brady, & Smith, 2001). The total volume of grey matter was calculated from these images (by multiplying the average voxel value by the total number of voxels). These images were also used in the functional analyses below as voxel-dependent covariates. For the VBM-style analyses of structure, the 32 images of grey matter were non-linearly registered to the MNI-152 grey matter template using FMRIB’s Nonlinear Registration Tool (FNIRT) (Andersson, Jenkinson, & Smith, 2007a, 2007b). Each image was flipped across the midline to create a mirror image and the 64 images were averaged to create a left–right symmetric study-specific grey matter template. The 32 original images of grey matter were then non-linearly transformed to this new template. The partial volume estimate in each voxel of the transformed images was modulated by the Jacobian determinant of the non-linear component of the warp field; that is, it was adjusted to reflect the extent each voxel was contracted or enlarged to match the template image. The resulting image contains voxels that represent the original volume of grey matter at each location for each subject. All 32 modulated and transformed grey matter images were smoothed with an isotropic Gaussian kernel with a sigma of 4 mm (∼10 mm full width at half maximum). Differences in grey matter volume were tested with independent t-tests between pairs of groups with age at scan and sex as covariates. Voxel-wise thresholds at *p* < 0.001 uncorrected were applied.

#### 2.3.3 Functional image analysis

Functional data from each individual were first analysed using fMRI Expert Analysis Tool (FEAT v5.98) running in FSL. The images were motion corrected by realignment to the middle volume of the 4D dataset, smoothed using a 6-mm full-width at half maximum smoothing kernel, and non-linearly registered via the participant’s T1-weighted structural image to the MNI-152 template. Low-frequency fluctuations were removed using a high-pass filter with a cutoff at 100 s. Image volumes that were outliers in terms of motion, and the motion correction parameters (translations and rotations in *x*, *y* and *z*) were included as covariates of no interest in the analyses. Statistical maps of activity corresponding to contrasts of the Speech and Reversed Speech conditions with the silent baseline and with each other were calculated using the general linear model. Group averages and differences between groups for each of these contrasts were calculated at a second-level analysis using FMRIB’s Local Analysis of Mixed Effects (FLAME) stage 1 (Woolrich, Behrens, Beckmann, Jenkinson, & Smith, 2004). The images of grey matter obtained in the structural analyses (see above) were transformed to the MNI-152 template and included as voxel-dependent covariates in the group analyses (Oakes et al., 2007). Peak locations for voxels with *Z* > 3.1 (*p* < 0.001, uncorrected) and comprising a cluster with 30 or more voxels are reported for group average contrasts.

Language lateralisation was assessed by calculating lateralisation indices (LI) for individual *z*-statistic images using the LI-toolbox (Wilke & Lidzba, 2007) run in SPM8. Based on our areas of interest, comprehensive frontal (excluding the medial wall using a 10 mm mask from the centre of the image) and temporal lobe standard LI-toolbox templates were used with a weighted-bootstrapping method of LI calculation (Wilke & Schmithorst, 2006). The LI formula used, LI = (*L* − *R*)/(*L* + *R*), results in positive values indicating left lateralisation and negative values, right lateralisation. Previous studies have adopted the convention of considering values between 0.2 and −0.2 as indicative of bilateral processing with values outside this range being indicative of left- or right-lateralised processing (Wilke et al., 2005, 2006).

## 3 Results

Individual scores and group medians for the behavioural tests are displayed in Table 1. The groups did not differ in their hand preference for writing, *χ*^2^(2) = 2.62, *p* = 0.27, and the SLI group scored significantly lower on all behavioural tests but one (hand preference demonstration) relative to the sibling and typical groups. These latter two groups did not differ (see Table 1).

### 3.1 Structural imaging

**Fig. 1:**
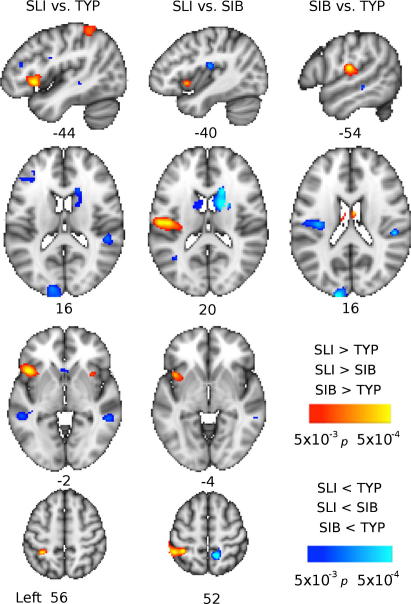
Group differences in grey matter volume revealed by voxel-based morphometry. Coloured maps show the *p*-value (thresholded at *p* < 0.005 for display purposes only) for comparisons between groups: Typical (TYP), Siblings (SIB), and SLI. Red–yellow areas have more grey matter in the SLI group compared to the SIB and TYP groups and the SIB group compared to the TYP group. Blue-light-blue areas have less grey matter in the SLI group compared to the SIB and TYP groups and the SIB group compared to the TYP group. Maps are presented on the standard MNI152 T1-weighted brain. Numbers below images indicate the coordinate in mm of that slice in *x* (for sagittal, top row) and *z* (for axial, second, third and bottom rows) relative to the orthogonal planes through the anterior commissure.

The total amounts of grey matter did not significantly differ between groups (means ± S.D.: SLI 749 ± 100 cm^3^; SIB 726 ± 76 cm^3^; TYP 738 ± 80 cm^3^). Voxel-wise comparisons revealed that the SLI group (*N* = 10) had significantly more grey matter than the Typical group (TYP, *N* = 16) in the left inferior frontal gyrus (IFG), right insula, and left intraparietal sulcus. They had significantly less grey matter than TYP in the posterior superior temporal sulcus (STS) bilaterally, extending to the superior temporal gyrus (STG) on the right, the right caudate nucleus and right side of the midbrain at the level of the substantia nigra, the medial frontal polar cortex, right medial superior parietal cortex and left occipital pole (see Fig. 1). Compared with their unaffected siblings (SIB, *N* = 6), the SLI group had significantly more grey matter in the left anterior intraparietal suclus and significantly less grey matter in the right parietal opercular cortex (and the left at a slightly lower statistical threshold) and left occipital pole (see Fig. 1). When the SIB group was compared with the TYP group, they had significantly more grey matter in the left central opercular cortex (ventral extent of the central sulcus) and the retrosplenial cortex bilaterally and significantly less grey matter in the caudate nucleus bilaterally, right putamen, right medial geniculate body and left fusiform gyrus (see Fig. 1). The peak locations and statistics associated with these peaks are summarised in Table 2.

In sum, the SLI group and their unaffected siblings showed reduced volume of the right caudate nucleus compared to typically developing controls; at lower statistical thresholds, the left caudate nucleus also showed reduced volume compared to controls for both SLI and SIB groups. The SLI group alone showed a striking abnormality in the left IFG, where they had significantly more grey matter than the TYP group. Conversely, they showed bilateral reductions in the grey matter of the posterior superior temporal cortex. As these are areas we expected to be activated in the functional task, we included grey matter volume estimates as voxel-wise covariates in the group-level functional data analysis. This ensured that any functional differences observed between groups were not due to these known differences in structure.

### 3.2 Functional imaging

**Fig. 2:**
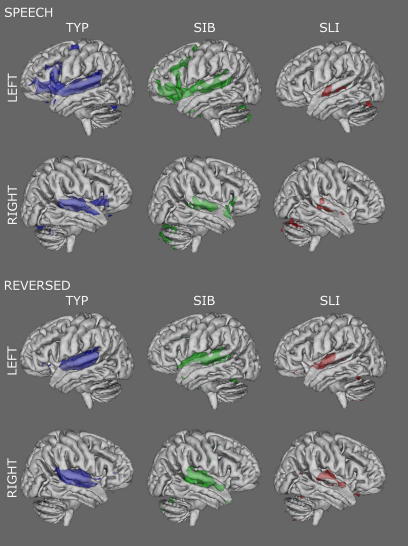
Group averages for the Speech and Reversed Speech contrasts with baseline. Three-dimensional statistical maps of left (top) and right (bottom) hemisphere activations are presented for Typically developing (TYP, purple), Siblings (SIB, green), and SLI (red) groups. Coloured activations are presented for the Speech (top) and Reversed Speech (bottom) conditions compared to baseline at a threshold of *Z* > 3.1 (*p* < 0.001, uncorrected) and above.

**Fig. 3:**
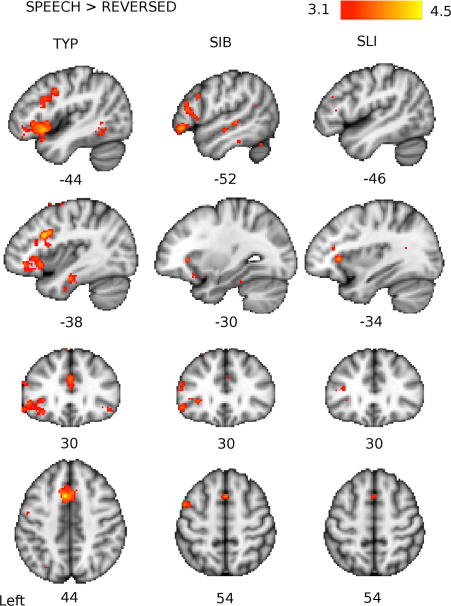
Group averages of brain activity for the Speech > Reversed contrast. Coloured maps show the *Z*-statistic (thresholded at *Z* > 3.1) for each group: Typical (TYP), Siblings (SIB), and SLI. Red-yellow areas have more activity in the Speech condition relative to the Reversed Speech condition. Maps are presented on the standard MNI152 T1-weighted brain. Numbers below images indicate the coordinate in mm of that slice in *x* (for sagittal, top two rows), *y* (for coronal, third row) and *z* (for axial, bottom row) relative to the orthogonal planes through the anterior commissure.

Group averages of activation for the Speech and Reversed conditions contrasted with the silent baseline are presented in Fig. 2. The anatomical location of statistical peaks, their MNI-space coordinates, *z*-statistics, and the extents of the cluster of voxels to which each is connected for the separate group analyses are presented in the Supplementary Tables. For Speech, analysis of data from the TYP group (*N* = 13) revealed activity in the expected network of brain regions involved in language processing. This included the left IFG, pre-supplementary motor area (preSMA), and extensive portions of the STG bilaterally. For Reversed Speech, the TYP group produced activation in regions associated with auditory processing namely bilateral activity along the STG. The contrast of Speech greater than Reversed Speech highlighted a clearly left-lateralised pattern of activation involving the left IFG and preSMA (see Fig. 3).

For the SIB group (*N* = 6), patterns of activation for all contrasts were similar to those seen in the TYP group (see Supplementary Tables for SIB activation descriptions); the extent of activations above the statistical threshold was somewhat reduced in the SIB compared to the TYP group, which may be due to the smaller number of participants in the former (*N* = 6) compared to the latter (*N* = 13). For the SLI group (*N* = 8), however, the extent of activity above the statistical threshold was severely reduced such that for Speech there were no supra-threshold voxels in the left IFG and the clusters of activity in the STG bilaterally were reduced in extent and the height of the statistic (see Supplementary Tables for SLI activation descriptions).

In sum, within-group patterns of activation for the three contrasts (see Figs. 2 and 3, and Supplementary Tables) are indicative of functionally similar patterns between all groups, suggesting that the groups did not differ in their general response to the conditions. However, the average intensity of activation did differ between groups, with activation in the SLI group mostly sub-threshold.^1^

### 3.3 Between-group comparisons

**Fig. 4:**
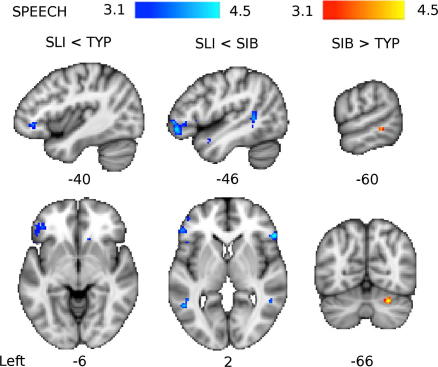
Group differences in brain activity for the Speech contrast with baseline. Coloured maps show the *Z*-statistic (thresholded at *Z* > 3.1) for comparisons between groups: Typical (TYP), Siblings (SIB), and SLI. Red–yellow areas have more activity in the SIB group compared to the TYP group. Blue-light-blue areas have less activity in the SLI group compared to the SIB and TYP groups. Maps are presented on the standard MNI152 T1-weighted brain. Numbers below images indicate the coordinate in mm of that slice in *x* (for sagittal, top row), *y* (for coronal, right image bottom row) and *z* (for axial, left and centre images bottom row) relative to the orthogonal planes through the anterior commissure.

**Fig. 5:**
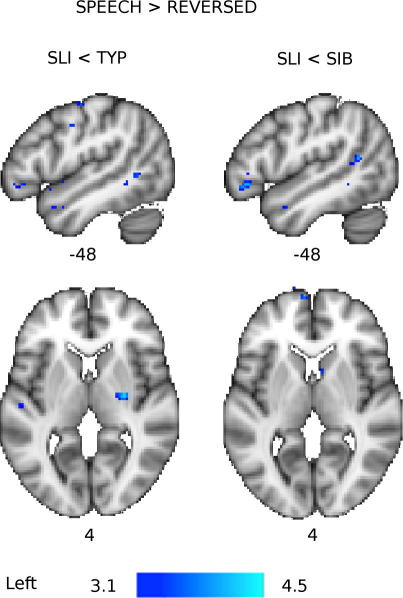
Group differences in brain activity for the Speech > Reversed contrast. Coloured maps show the *Z*-statistic (thresholded at *Z* > 3.1) for comparisons between groups: Typical (TYP), Siblings (SIB), and SLI. Blue-light-blue areas have less activity in the SLI group compared to the SIB and TYP groups. Maps are presented on the standard MNI152 T1-weighted brain. Numbers below images indicate the coordinate in mm of that slice in *x* (for sagittal, top row) and *z* (for axial, bottom row) relative to the orthogonal planes through the anterior commissure.

The differences in patterns of activation among the three groups described above were tested directly by statistical contrasts between them. Compared to the TYP group, the SLI group had significantly reduced activity in the left IFG (pars orbitalis) during the Speech condition (see Fig. 4) and in the left STG and right putamen for the contrast of Speech greater than Reversed (see Fig. 5 and Table 3 for all between-group comparisons). Activity in the SLI group was also reduced relative to the TYP group in the left IFG for the Speech greater than Reversed contrast; however, this difference did not pass our inclusion criterion with an extent of only 8 voxels. Compared to the SIB group, the SLI group had significantly reduced activity in the IFG and STG bilaterally for both the Speech and the Speech greater than Reversed Speech contrasts (see Figs. 4 and 5). Overall, these results indicate a reduced speech-specific response in this SLI group.

The comparison of the SIB and TYP groups revealed greater activation in the SIB group in the right cerebellar lobule VI during the Speech condition (see Fig. 4 and Table 3). There were no significant differences between the SIB and TTP groups in the other contrasts.

There were no significant group differences in the Reversed Speech contrast.

### 3.4 Lateralisation

**Fig. 6:**
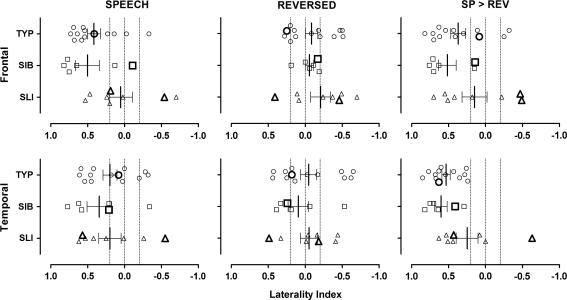
Laterality indices for functional activation in the frontal and temporal lobes. Data for individual participants are shown for Typical (TYP: circles), Sibling (SIB: squares), and Specific Language Impairment (SLI: triangles) groups for frontal (top row) and temporal lobes (bottom row) for the Speech, Reversed Speech (Reversed), and Speech greater than Reversed (Sp > Rev) contrasts. Error bars represent the standard error of the mean and the *x*-axis has been reversed so that laterality indices to the left of centre represent left lateralisation and values to the right represent right lateralisation. Additional lines have been placed at 0.2 and −0.2 as an indication of divisions for left, bilateral, and right lateralisation (see text). Left-handed individuals’ data points are bolded.

Laterality indices based upon the frontal and temporal lobes for the three contrasts are presented in Fig. 6. The results for the TYP and SIB groups show laterality patterns consistent with performance of a language task: (1) left-lateralised activity was seen in the frontal lobes for Speech contrasted with either baseline or Reversed Speech conditions; (2) left-lateralised activity was also seen in the temporal lobes when Speech was contrasted with Reversed Speech but not when either was contrasted with baseline; (3) the activity for Reversed Speech was not lateralised in either frontal or temporal lobes.

The pattern in the SLI group was less lateralised in both frontal and temporal lobes for the Speech greater than Reversed Speech contrast (see Fig. 6). This was mainly due to three individuals in the SLI group who showed a tendency to right lateralisation (two) or no clear lateralisation (one). The individual in the SLI group who was most clearly right lateralised was also left-handed. There was a significant difference between the SLI and TYP groups in the laterality indices for frontal lobe activation for the Speech condition only; SLI vs. TYP, *U* = 22, *p* = 0.03, *r* = −0.47; SLI vs. SIB, *U* = 11, *p* = 0.09, *r* = −0.45.

### 3.5 Relating structural and functional abnormalities

The SLI group showed both structural and functional abnormalities in several areas. The left inferior frontal cortex showed increased grey matter and decreased functional activation, whereas the posterior temporal cortex showed both decreased grey matter and functional activation. Grey matter volume estimates and percent signal change for the Speech condition were extracted for each participant at the first-level from 6-mm radius spherical regions of interest centred on the coordinates reported in Table 2. Also, because previous studies in the KE family had noted reduced grey matter in the caudate nucleus and found this to be related to behavioural measures on nonword repetition and oromotor praxis (see Watkins et al., 2002b), we examined the same correlations in the SLI and the SIB groups separately. These analyses showed a negative correlation between nonword repetition and grey matter volume in the right caudate nucleus for the SLI group (*ρ* = −0.55, *p* = 0.05); the remaining correlations were not significant.

## 4 Discussion

We compared brain structure and function during a language task in a group of individuals with SLI, their unaffected siblings and typically developing controls. The SLI group had significantly more grey matter than controls in the left inferior frontal gyrus (IFG) and significantly less grey matter in the right caudate nucleus and the superior temporal sulcus (STS) bilaterally. Functionally, when performance of the covert naming task was contrasted with a silent baseline or passive listening to reversed speech, the SLI group showed generally reduced activity relative to the sibling and typical groups. This underactivity was localised to the left IFG, the right putamen, and to the STS/G bilaterally. Furthermore, lateralisation, clearly left in the sibling and typical groups, was reduced in the SLI group. There were no areas where the SLI group showed activation greater than the sibling or typical groups, which might have been interpreted as evidence for different functional organisation for language or compensatory or maladaptive reorganisation.

The finding of both structural and functional abnormalities in the left IFG and posterior temporal cortex bilaterally is consistent with the known roles these regions play in language; damage to one or more of these regions acquired in adulthood gives rise to different forms of aphasia. The relationships between the structural and functional abnormalities seen in our study differed in the frontal and temporal regions, however. In the frontal region (Broca’s area), grey matter was abnormally increased in SLI, whereas functional activation was reduced; these differences were seen both in comparison with controls and with unaffected siblings. In the posterior temporal cortex (Wernicke’s area), however, both the amount of grey matter and the amount of functional activation were reduced in SLI. Even though the SLI group showed these spatially coincident abnormalities in structure and function, within the group, grey matter volume and percentage signal change in each of these brain regions were not correlated.

The correspondence between the findings reported here for SLI and previous findings in the KE family is striking. Affected members of the KE family show a behavioural profile very similar to that seen in SLI (Watkins et al., 2002a). Relevant here is that imaging studies show the affected members of the KE family also had increased grey matter in the left IFG (Watkins et al., 2002b) and reduced functional activity in this region during verb generation and word repetition (Liégeois et al., 2003), which is the same as the pattern of structural and functional abnormalities we see here in SLI.

The most robust grey matter abnormality found in the KE family was a reduction in the volume of the caudate nucleus bilaterally; in affected family members the right caudate nucleus volume was significantly negatively correlated with nonword repetition, whereas the left caudate nucleus volume was significantly positively correlated with oromotor praxis (Watkins et al., 2002b). In our study of SLI, the right caudate nucleus was significantly reduced in grey matter volume compared to controls; the left nucleus also had less grey matter in SLI but this difference was not significant at the threshold used. We also replicated Watkins et al.’s finding of a negative correlation between nonword repetition and right caudate nucleus volume in the SLI group, despite using a different behavioural test and method of analysis of grey matter volume estimation. Functionally, another part of the striatum, the putamen, was found to be underactive in our study of SLI and in the affected members of the KE family (Liégeois et al., 2003). The striatum has been related to preparatory motor control (Duffau, 2008; Grahn, Parkinson, & Owen, 2008; Ketteler, Kastrau, Vohn, & Huber, 2008). Reductions in caudate nucleus volume have previously been associated with language impairment (Jernigan et al., 1991; Tallal, Jernigan, & Trauner, 1994).

Although the correspondence between the two sets of studies is impressive the pattern of abnormalities in SLI also differs from that seen in the KE family in several ways. In the current study, grey matter in the posterior temporal cortex in SLI is significantly decreased relative to controls, whereas it was increased in affected KE family members. Similarly, the putamen was found to have increased grey matter in affected KE family members, whereas we found no structural differences in the putamen in SLI. Finally, the caudate nucleus was found to be significantly reduced in volume in affected KE family members relative to their unaffected relatives and functionally overactive in a PET study of word repetition (Watkins et al., 1999). In our SLI study and the functional MRI study of the KE family, the caudate nucleus was not reliably activated by the task used and no group differences in function were detected. Also, the unaffected siblings in our study had significantly less grey matter in the caudate nucleus bilaterally relative to the typically developing controls and did not differ significantly from their siblings with SLI. The latter suggests that reduced caudate nucleus volume can be considered a heritable risk factor for SLI but requires additional deficits to affect language development. Alternatively, the siblings in our study have some protective factors, plasticity or compensatory mechanisms available to them that are unavailable to their affected siblings. The increased grey matter of the left central opercular cortex in the unaffected siblings relative to the SLI and control groups might reflect such compensatory mechanisms.

The similarities between the functional and structural abnormalities in this group of people with SLI and the affected members of the KE family are likely a reflection of the similarities in their behavioural deficits. Both groups have impairments in nonword repetition and oromotor function. Whether any of the individuals with SLI that we studied also have a mutation in *FOXP2* is unknown, but is unlikely, given the rarity of such mutations in individuals with SLI (Newbury & Monaco, 2010). In a larger population of SLI, however, allelic variation in a downstream target gene of FOXP2, *CNTNAP2* was found to correlate with performance on nonword repetition (Vernes et al., 2008), so investigations of this gene in our participants are warranted.

Previous developmental studies measuring grey matter volume and cortical thickness have revealed gradual linear and nonlinear reductions in these measures that continue into the second decade (e.g., Giedd et al., 1999; Giorgio et al., 2010; Gogtay et al., 2004). These changes are commonly interpreted as reflecting the normal maturation process within cortex that involves initially an overproliferation of synapses followed by elimination due to axonal pruning (e.g., Huttenlocher & Dabholkar, 1997). One interpretation of our finding of increased grey matter in the left posterior IFG (i.e., Broca’s area) in SLI is that cortex in this region has not undergone the normal maturation processes at the same rate as in the sibling or typical groups. Whether this is the cause of the lack of functional specialisation (and activation) of this area, or a consequence of it, remains uncertain.

In typical development, the IFG is linked with the STS/G via at least two streams that are important for auditory language processing in the left hemisphere (Rauschecker & Scott, 2009). In our study of SLI, the reduced grey matter and reduced activity in the STS/G occurred bilaterally and was specific to language processing and not more general auditory processes, given similar between group activations in the Reversed Speech condition. Regular firing of neural pathways leads to strengthening, maintenance, and building of connections, so reductions in volume to the STS/G may derive from underactivity in this area (synaptic elimination; Huttenlocher & Dabholkar, 1997), potentially driven by a system that is less stimulated by speech specific stimuli. Alternatively, a causal hypothesis is that experience has not altered the cortex and that less grey matter in the STS/G underpins the language difficulties. Longitudinal investigations have been informative regarding other developmental disorders and could help distinguish these possibilities (Giedd & Rapoport, 2010).

The patterns of activation in the SLI group are more heterogeneous relative to both the unaffected siblings and typical groups. This is clearly visible in the laterality indices (see Fig. 6) with a greater number of SLI individuals demonstrating atypical lateralisation (i.e., more bilateral to rightward). This is consistent with the majority of existing research (Bernal & Altman, 2003; Chiron et al., 1999; Lou et al., 1990; Ors et al., 2005; Shafer, Schwartz, Morr, Kessler, & Kurtzberg, 2000; Whitehouse & Bishop, 2008) and suggests that the reduced activity noted at the group level is not the defining feature. It is worth noting that only one SLI participant shows reliably right-lateralised speech for the comparison of Speech with baseline and with Reversed Speech and for both the frontal and the temporal lobe areas considered. Another left-handed participant with SLI shows more left-lateralised activation for Reversed Speech than Speech resulting in a rightwards LI for the Speech contrast with Reversed Speech. Finally, a few of the right-handed controls (TYP and SIB) and one right-handed individual with SLI also show a pattern of rightwards lateralisation. Further research is needed to examine whether the increased variability in SLI is also seen from stimulus to stimulus or session to session.

Our implementation of the covert naming task was designed to be easy so that all participants could provide equivalent behavioural responses. This was verified before scanning with a small subset of stimuli used by the experimenter to demonstrate the task and another set of practice items. However, the specific ease with which particular participants or groups completed the task during scanning is unknown and may be variable. Variations in task difficulty can affect physiological responses, linearly increasing neuronal firing with increasing difficulty (Chen et al., 2008) and increasing amplitude of electrical activity (Mulert et al., 2007). However, using functional transcranial Doppler ultrasound, we have shown that difficulty in both an auditory naming and a word generation task does not affect lateralisation or the intensity of activation (Badcock, Nye, & Bishop, in press).

There are a number of limitations of this research that relate to the small sample size and differences between the groups in terms of age ranges and distribution of handedness and sex. Although the group sizes are small, they are comparable with group sizes from other studies of brain structure and function in language-impaired populations (e.g., Watkins et al., 2002b). To minimise the effects of differences on brain structure relating to factors such as age, sex and handedness, we implemented the use of a nonlinear registration of the functional images to standard space, which removes gross differences in size and shape among the brains. We also included an image of grey matter volume for each individual subject as a voxel-dependent covariate in the functional analysis; only functional differences over and above structural differences would remain, therefore. Finally, although our groups were small, we used a mixed-effects analysis to compare groups rather than a fixed-effects analysis, which is typically used in small samples of special populations. By using a mixed-effects analysis, which combines between-subject and within-subject variance at the group level, our data are less likely to be influenced by outliers, such as the left-handed SLI subject whose LI is reliably right-lateralised. This approach allows us to generalise our results to the wider population rather than limit their inference to the study-population as with a fixed-effects analysis. In our experience, brain structure is minimally affected by handedness and sex (see Watkins et al., 2001), so the age differences among our participants is likely to be the main confound. It is well described that although white matter continues to increase linearly across the life span, grey matter increases to a peak during childhood or adolescence and then decreases during later years (Giedd et al., 1999; Gogtay et al., 2004). A longitudinal analysis of grey matter volume collected on the same scanner with the same protocol as used here and analysed with the same tools, revealed reductions in grey matter from in a cohort aged 13 to 19 year olds over a 2–3 year period in mainly right hemisphere regions (Giorgio et al., 2010); no age-related reductions were seen in this time and in this age range in the left inferior frontal and superior temporal regions that showed structural and functional differences in the SLI group. We feel that it is unlikely that the structural and functional differences in these regions between the SLI group and the other two groups are due to age differences, but further study using larger samples is warranted.

## 5 Conclusion and future directions

The structural and functional investigations into SLI provide useful insights into the neural differences which may underpin the language difficulties observed behaviourally. There is clear evidence of atypical structure and function in the left inferior frontal and superior temporal areas known to be involved in language production and comprehension. Subcortical components including the caudate nucleus and putamen are also implicated, most likely due to their involvement with motor response planning, selection, and preparation. Future investigations should aim to elucidate the developmental trajectories of structure and function, functionally assessing both receptive and expressive components independently. Between-group consideration of the task demands may also be important, attempting to minimise any influence of task difficulty. Furthermore, considering both left and right hemisphere specialisation and organisation, assessing prosodic speech aspects and regional connections will provide useful insights.

## Figures and Tables

**Table 1:** Demographics and behavioural test scores for all participants. Data are shown for all individuals and the median scores were calculated for the SLI, SIB and TYP groups separately.

Scores are standard scores (mean 100 ± 15) unless otherwise indicated. Shaded and bolded group medians indicate a significantly higher median for the TYP or SIB group relative to the SLI group (Mann–Whitney *U* test, *p* < 0.015 using B–H method FDR; (Narum, 2006). ^a^Number of actions demonstrated with the right hand (out of a maximum of 10). ^b^Scores for the General Communication Composite obtained with the age appropriate Communication Checklist. Scores greater than 58 are considered normal. ^c^Scores represent percentile categorisations: 1 = <2nd, 2 = 2nd–10th, 3 = 10–25th, 4 = 25–75th, 5 = >75th.

**Table 2:** Results of group comparisons for grey matter volume using voxel-based morphometry.

Anatomical region	*X*	*Y*	*Z*	*t*	*p*
*SLI more grey than TYP*					
Left frontal operculum	−44	18	−2	5.17	<0.0001
Right anterior insula	34	14	−2	3.46	0.0010
Left anterior intraparietal sulcus	−36	−44	56	4.12	0.0002

*SLI less grey than TYP*					
Medial frontal pole	−6	60	−16	4.53	<0.0001
Right Caudate Nucleus (head)	16	20	16	3.55	0.0008
Right substantia nigra	10	−18	−14	3.63	0.0006
Right posterior STG	50	−36	14	4.06	0.0002
Right posterior MTG	56	−38	−6	3.85	0.0004
Left posterior STS	−54	−38	−2	3.49	0.0009
Right medial superior parietal	12	−50	56	4.82	<0.0001
Left occipital pole	−12	−94	16	3.96	0.0003

*SLI more grey than SIBs*					
Left anterior intraparietal sulcus	−32	−44	52	4.05	0.0005

*SLI less grey than SIBs*					
Left parietal operculum^a^	−36	−18	20	3.72	0.0010
Right parietal operculum	50	−28	20	3.94	0.0007
Left occipital pole	−14	−100	20	4.43	0.0002

*SIBs more grey than TYP*					
Left central operculum	−54	−18	16	4.04	0.0003
Right retrosplenial cortex	6	−42	2	4.29	0.0002
Left retrosplenial cortex^a^	−4	−48	2	3.54	0.0010

*SIBs less grey than TYP*					
Right caudate nucleus (head)	14	10	22	4.55	<0.0001
	20	2	16	5.16	<0.0001
Right putamen	20	4	12	4.29	0.0002
Left caudate nucleus (head)^∗^	−12	0	12	3.25	0.0019
Right medial geniculate body	26	−20	−6	3.71	0.0006
Left posterior fusiform gyrus	−26	−74	−8	3.72	0.0006

Results are reported for areas that survive a statistical threshold of *p* < 0.001 in each between-group contrast, except for regions highlighted with ∗, which were not significant at this threshold but were symmetrical with a region that was. *X*, *Y* and *Z* are coordinates in the standard space of the MNI-152 T1-weighted template, *t* is the *t*-statistic, *p* is the *p*-value of the *t*-statistic; note that this differs for different group contrasts because of the degrees of freedom.

**Table 3:** Results of group comparisons for functional activation during covert auditory naming.

Contrast	Brain area	*X*	*Y*	*Z*	z-Statistic	Voxels
*Less activity in SLI than TYP*						
Speech	Left inferior frontal gyrus, pars orbitalis	−40	38	−4	3.84	102
Sp > Rev	Right putamen	28	−12	4	4.29	95
	Left superior temporal gyrus, posterior	−62	−50	20	3.99	34

*Less activity in SLI than SIB*						
Speech	Left inferior frontal gyrus, pars orbitalis	−46	44	−8	4.33	248
	Right inferior frontal gyrus, pars triangularis	56	28	2	4.65	52
	Left superior temporal sulcus, posterior	−48	−48	8	4.45	131
Sp > Rev	Left inferior frontal gyrus, pars orbitalis	−48	32	−6	4.11	35

*More activity in SIB than TYP*						
Speech	Right cerebellar lobule VI	26	−66	−28	4.19	31

Between group contrasts activation for Speech against baseline and Speech greater than Reversed Speech (Sp > Rev). Differences are significant at *Z* > 3.1 and with extents of 30 or more voxels. Brain locations are presented for *X* (sagittal), *Y* (coronal) and *Z* (axial) coordinates in mm relative to the orthogonal planes through the anterior commissure, together with peak *z*-statistic, and extent size in voxels. There were no significant between group differences in activation for the Reversed Speech contrast.

## Appendix A Supplementary material

Supplementary Table S1 Typical group activation for Speech and Reversed Speech (Reversed) against the silent baseline and Speech greater than Reversed Speech.

Supplementary Table S2 Sibling group activation for Speech and Reversed Speech (Reversed) against the silent baseline and Speech greater than Reversed Speech.

Supplementary Table S3 Specific language impairment group activation for Speech and Reversed Speech (Reversed) against the silent baseline and Speech greater than Reversed Speech.

## Footnotes

1 The absolute number of active voxels was significantly above zero in all groups (Wilcoxon Signed Ranks, *p* < 0.05).
