# Supplementary material for: Co-localisation of abnormal brain structure and function in specific language impairment
Source: Brain Lang. 2012 Mar;120(3-4):310–20. doi: 10.1016/j.bandl.2011.10.006 (PMC3315677; doi:10.1016/j.bandl.2011.10.006)
Supplement: Supplementary Table S1 — Typical group activation for Speech and Reversed Speech (Reversed) against the silent baseline and Speech greater than Reversed Speech. [file mmc1.doc]

Supplementary Table 1 Typical group activation for Speech and Reversed Speech (Reversed) against the silent baseline and Speech greater than Reversed Speech.

Differences are significant at Z > 3.1 and with extents of 30 or more voxels. Brain locations are presented for X (sagittal), Y (coronal) and Z (axial) coordinates in mm relative to the orthogonal planes through the anterior commissure, together with peak z-statistic, and extent size in voxels. note: for clusters extending over more than one lobe, the peak and local maxima are reported.

| Contrast | Brain Area | X | Y | Z | z-statistic | voxels |
| --- | --- | --- | --- | --- | --- | --- |
| Speech | Right superior temporal gyrus, anterior | 40 | 28 | -28 | 4.1 | 35 |
|  | Left putamen | -18 | 10 | -2 | 5.1 | 173 |
|  | Left posterior orbital gyrus | -28 | 10 | -24 | 4.24 | 38 |
|  | Left superior frontal gyrus | -2 | 8 | 48 | 5.37 | 1085 |
|  | Right head of caudate nucleus | 16 | 8 | 8 | 4.39 | 120 |
|  | Left inferior temporal gyrus | -40 | -10 | -38 | 3.78 | 35 |
|  | Left thalamus | -8 | -14 | 4 | 4.18 | 55 |
|  | Left superior temporal gyrus, posterior | -62 | -24 | 2 | 7.35 | 6649 |
|  | Left insula | -30 | 24 | 0 | 6.63 |  |
|  | Left superior temporal sulcus, medial | -54 | -14 | -6 | 6.6 |  |
|  | Left inferior frontal gyrus, pars triangularis | -42 | 30 | -2 | 6.51 |  |
|  | Right superior temporal gyrus, posterior | 50 | -28 | 4 | 6.79 | 3115 |
|  | Right inferior frontal gyrus, pars opercularis and triangularis | 54 | 12 | -4 | 5.7 |  |
|  | Left inferior temporal gyrus | -48 | -56 | -8 | 3.92 | 70 |
|  | Left calcarine sulcus | -24 | -64 | 4 | 4.54 | 140 |
|  | Right cerebellar crus II | 44 | -64 | -30 | 3.51 | 46 |
|  | Left superior lingual gyrus | -4 | -64 | 0 | 4.07 | 34 |
|  | Right cerebellar lobule VII | 32 | -66 | -56 | 4.51 | 65 |
|  | Right cerebellar crus II | 12 | -82 | -40 | 5.39 | 355 |
| Reversed | Right inferior frontal gyrus, pars triangularis | 56 | 34 | 2 | 4.18 | 53 |
|  | Left inferior frontal gyrus, pars triangularis | -42 | 30 | -2 | 4.15 | 64 |
|  | Rright inferior frontal gyrus, pars opercularis | 56 | 20 | 10 | 3.95 | 38 |
|  | Right superior temporal gyrus, posterior to anterior | 56 | -14 | 4 | 6.87 | 3086 |
|  | Left superior temporal gyrus, posterior to anterior | -60 | -24 | 2 | 7.54 | 3161 |
| Sp > Rev | Right superior frontal sulcus | 30 | 48 | 24 | 4.15 | 31 |
|  | Left superior frontal sulcus | -28 | 46 | 22 | 4.24 | 50 |
|  | Right inferior frontal gyrus | 46 | 20 | -6 | 5.82 | 514 |
|  | Left inferior frontal gyrus/sulcus, pars opercularis | -38 | 16 | 28 | 5.72 | 375 |
|  | Left medial frontal pre-supplementary motor area | -6 | 14 | 44 | 5.92 | 1229 |
|  | Right cingulate sulcus | 10 | 22 | 30 | 5.47 |  |
|  | Left superior frontal pre-supplementary motor area | -6 | 8 | 56 | 5.26 |  |
|  | Right putamen | 16 | 4 | -10 | 4.29 | 41 |
|  | Left superior temporal sulcus, anterior | -60 | -2 | -12 | 6.04 | 2386 |
|  | Left middle temporal gyrus, posterior | -48 | -54 | -8 | 5.34 |  |
|  | Left insula | -32 | 26 | 2 | 5.09 |  |
|  | Right globus pallidus | 20 | -6 | -4 | 3.98 | 36 |
|  | Left thalamus | -2 | -8 | 10 | 3.87 | 41 |
|  | Left globus pallidus | -14 | -10 | -4 | 4.03 | 31 |
|  | Left fusiform gyrus | -38 | -16 | -24 | 4.26 | 82 |
|  | Left collateral sulcus | -32 | -26 | -22 | 3.83 | 43 |
|  | Left cerebellar crus I | -52 | -60 | -26 | 4.18 | 38 |
|  | Calcarine sulcus | -24 | -66 | 4 | 4.75 | 278 |
|  | Right cerebellar lobule VI | 4 | -80 | -18 | 4.81 | 133 |
