# Supplementary material for: Co-localisation of abnormal brain structure and function in specific language impairment
Source: Brain Lang. 2012 Mar;120(3-4):310–20. doi: 10.1016/j.bandl.2011.10.006 (PMC3315677; doi:10.1016/j.bandl.2011.10.006)
Supplement: Supplementary Table S2 — Sibling group activation for Speech and Reversed Speech (Reversed) against the silent baseline and Speech greater than Reversed Speech. [file mmc2.doc]

Supplementary Table 2 Sibling group activation for Speech and Reversed Speech (Reversed) against the silent baseline and Speech greater than Reversed Speech.

Differences are significant at Z > 3.1 and with extents of 30 or more voxels. Brain locations are presented for X (sagittal), Y (coronal) and Z (axial) coordinates in mm relative to the orthogonal planes through the anterior commissure, together with peak z-statistic, and extent size in voxels. note: for clusters extending over more than one lobe, the peak and local maxima are reported.

| Contrast | Brain Area | X | Y | Z | z-statistic | voxels |
| --- | --- | --- | --- | --- | --- | --- |
| Speech | Left inferior frontal gyrus, pars triangularis | -54 | 34 | -6 | 5.55 | 4753 |
|  | Left superior temporal gyrus, posterior | -62 | -28 | 2 | 5.53 |  |
|  | Left superior Frontal gyrus | -16 | 32 | 46 | 4.33 | 198 |
|  | Right inferior frontal gyrus, pars triangularis | 56 | 28 | 2 | 5.16 | 445 |
|  | Right middle frontal gyrus | 44 | 6 | 44 | 4.11 | 33 |
|  | Left medial frontal pre-supplementary motor area | -2 | 4 | 62 | 4 | 85 |
|  | Left precentral gyrus | -46 | 0 | 58 | 4.3 | 108 |
|  | Left putamen | -22 | -4 | 6 | 3.95 | 50 |
|  | Right superior temporal gyrus, posterior | 50 | -22 | 2 | 5.2 | 1140 |
|  | Left middle/inferior temporal gyrus, deep | -44 | -42 | -10 | 4.02 | 48 |
|  | Left fusiform gyrus | -40 | -44 | -20 | 4.17 | 32 |
|  | Right cerebellar Lobule VI | 26 | -66 | -28 | 5.52 | 1094 |
|  | Right cerebellar crus I | 42 | -66 | -32 | 4.52 | 41 |
|  | Left cerebellar crus II | -28 | -86 | -36 | 3.94 | 42 |
| Reversed | Right middle temporal gyrus, anterior | 42 | 14 | -36 | 3.82 | 67 |
|  | Left superior temporal gyrus, anterior | -46 | 12 | -26 | 3.68 | 31 |
|  | Left superior temporal sulcus/gyrus, anterior | -40 | 4 | -20 | 5.17 | 59 |
|  | Left superior temporal gyrus, posterior to anterior | -62 | -20 | 2 | 5.88 | 2164 |
|  | Right superior temporal gyrus, posterior to anterior | 64 | -28 | 6 | 6.25 | 2523 |
|  | Left cerebellar lobule VI | -28 | -66 | -24 | 4.96 | 87 |
|  | Left cerebellar lobule VIIIa | -24 | -66 | -52 | 3.76 | 40 |
|  | Right cerebellar lobule VIIIa | 26 | -66 | -54 | 3.81 | 36 |
|  | Right cerebellar crus I | 28 | -68 | -28 | 4.6 | 89 |
|  | Left cerebellar crus II | -20 | -82 | -38 | 4.04 | 46 |
| Sp > Rev | Left inferior frontal gyrus, pars orbitalis/pars triangularis | -52 | 34 | -8 | 4.97 | 358 |
|  | Left anterior insula | -30 | 28 | 2 | 4.17 | 52 |
|  | Left middle frontal gyrus | -48 | 14 | 32 | 4.71 | 71 |
|  | Right superior frontal gyrus | 2 | 12 | 54 | 4.48 | 50 |
|  | Left superior frontal gyrus | -2 | 4 | 60 | 3.95 | 86 |
|  | Left precentral gyrus | -48 | 2 | 54 | 4.48 | 52 |
|  | Left middle temporal gyrus, posterior | -50 | -28 | -2 | 4.77 | 105 |
|  | Right cerebellar crus I, medial | 12 | -78 | -30 | 4.21 | 43 |
|  | Right cerebellar crus I, lateral | 28 | -88 | -32 | 3.76 | 44 |
|  | Right cerebellar crus II, lateral | 6 | -90 | -38 | 3.94 | 47 |
