# Supplementary material for: Co-localisation of abnormal brain structure and function in specific language impairment
Source: Brain Lang. 2012 Mar;120(3-4):310–20. doi: 10.1016/j.bandl.2011.10.006 (PMC3315677; doi:10.1016/j.bandl.2011.10.006)
Supplement: Supplementary Table S3 — Specific language impairment group activation for Speech and Reversed Speech (Reversed) against the silent baseline and Speech greater than Reversed Speech. [file mmc3.doc]

Supplementary Table 3 Specific Language Impairment group activation for Speech and Reversed Speech (Reversed) against the silent baseline and Speech greater than Reversed Speech.

| Contrast | Brain Area | X | Y | Z | z-statistic | voxels |
| --- | --- | --- | --- | --- | --- | --- |
| Speech | Left inferior frontal gyrus, pars triangularis | -50 | 28 | 14 | 4.15 | 62 |
|  | Left superior frontal gyrus | -2 | 6 | 48 | 4.64 | 240 |
|  | Right superior temporal gyrus, anterior | 56 | 4 | -10 | 3.77 | 32 |
|  | Left thalamus, anterior thalamic nucleus | -12 | -10 | 14 | 3.97 | 32 |
|  | Left & right thalamus | 0 | -12 | 6 | 4.3 | 80 |
|  | Left superior temporal gyrus, posterior | -58 | -16 | 2 | 5.35 | 502 |
|  | Right superior temporal gyrus, posterior | 48 | -26 | 2 | 5.06 | 573 |
|  | Left superior temporal sulcus | -44 | -32 | -2 | 3.64 | 45 |
|  | Right cerebellar lobule VI | 28 | -64 | -26 | 4.15 | 218 |
|  | Left vermis VIII | -2 | -64 | -40 | 4.12 | 54 |
|  | Right superior lingual gyrus | 6 | -66 | -8 | 4.72 | 62 |
|  | Right vermis VI | 4 | -76 | -18 | 4.16 | 98 |
| Reversed | Right superior temporal gyrus, anterior | 40 | 26 | -26 | 4.17 | 37 |
|  | Right superior temporal gyrus, anterior | 48 | 10 | -16 | 4.11 | 36 |
|  | Left superior temporal gyrus (Heschl’s) | -58 | -16 | 2 | 6.19 | 1199 |
|  | Right superior temporal gyrus (planum temporale) | 50 | -26 | 6 | 5.67 | 1163 |
|  | Right cerebellar lobule VI | 24 | -60 | -22 | 4.16 | 52 |
|  | Left cerebellar lobule VI | -28 | -62 | -22 | 4.01 | 50 |
|  | Right cerebellar lobule V | 6 | -64 | -8 | 4.32 | 53 |
|  | Right cerebellar lobule VIIb | 22 | -74 | -58 | 4.27 | 59 |
| Sp > Rev | Left inferior frontal gyrus, pars triangularis | -34 | 30 | 16 | 3.62 | 14 |
|  | Left anterior insula | -34 | 26 | 2 | 4.92 | 52 |
|  | Left medial frontal pre-supplementary motor area | 0 | 12 | 54 | 3.67 | 29 |
|  | Right medial frontal pre-supplementary motor area | 8 | 6 | 66 | 3.43 | 11 |
|  | Right cerebellar crus II | 34 | -74 | -48 | 4.08 | 16 |
|  | Right cerebellar crus I | 16 | -84 | -22 | 3.89 | 17 |

Differences are significant at Z > 3.1 and with extents of 30 or more voxels for the Speech and Reversed comparisons, and 10 or more for the Speech > Reversed comparison. Brain locations are presented for X (sagittal), Y (coronal) and Z (axial) coordinates in mm relative to the orthogonal planes through the anterior commissure, together with peak z-statistic, and extent size in voxels.
